# The citrate transporter SLC13A5 as a therapeutic target for kidney disease: evidence from Mendelian randomization to inform drug development

**DOI:** 10.1186/s12916-023-03227-5

**Published:** 2023-12-18

**Authors:** Dipender Gill, Loukas Zagkos, Rubinder Gill, Thomas Benzing, Jens Jordan, Andreas L. Birkenfeld, Stephen Burgess, Grit Zahn

**Affiliations:** 1 Department of Epidemiology and Biostatistics, School of Public Health, Imperial College London, London, UK; 2 Primula Group Ltd, London, UK; 3 Department II of Internal Medicine and Center for Molecular Medicine Cologne (CMMC), University of Cologne, Faculty of Medicine and University Hospital Cologne, Cologne, Germany; 4 Cologne Excellence Cluster On Cellular Stress Responses in Aging-Associated Diseases (CECAD), University of Cologne, Cologne, Germany; 5 Institute of Aerospace Medicine, German Aerospace Center (DLR), Cologne, Germany; 6 Medical Faculty, University of Cologne, Cologne, Germany; 7 Department of Diabetology Endocrinology and Nephrology, Internal Medicine IV, University Hospital Tübingen, Eberhard Karls University Tübingen, Tübingen, Germany; 8 Division of Translational Diabetology, Institute of Diabetes Research and Metabolic Diseases (IDM) of the Helmholtz Center Munich, Eberhard Karls University Tübingen, Tübingen, Germany; 9 Department of Diabetes, School of Life Course Science and Medicine, King’s College London, London, UK; 10 Medical Research Council Biostatistics Unit at the University of Cambridge, Cambridge, UK; 11 Eternygen GmbH, Berlin, Germany

**Keywords:** SLC13A5, Citrate, Kidney, Renal function, Mendelian randomization, Drug development

## Abstract

**Background:**

Solute carrier family 13 member 5 (SLC13A5) is a Na^+^-coupled citrate co-transporter that mediates entry of extracellular citrate into the cytosol. SLC13A5 inhibition has been proposed as a target for reducing progression of kidney disease. The aim of this study was to leverage the Mendelian randomization paradigm to gain insight into the effects of SLC13A5 inhibition in humans, towards prioritizing and informing clinical development efforts.

**Methods:**

The primary Mendelian randomization analyses investigated the effect of SLC13A5 inhibition on measures of kidney function, including creatinine and cystatin C-based measures of estimated glomerular filtration rate (creatinine-eGFR and cystatin C-eGFR), blood urea nitrogen (BUN), urine albumin-creatinine ratio (uACR), and risk of chronic kidney disease and microalbuminuria. Secondary analyses included a paired plasma and urine metabolome-wide association study, investigation of secondary traits related to SLC13A5 biology, a phenome-wide association study (PheWAS), and a proteome-wide association study. All analyses were compared to the effect of genetically predicted plasma citrate levels using variants selected from across the genome, and statistical sensitivity analyses robust to the inclusion of pleiotropic variants were also performed. Data were obtained from large-scale genetic consortia and biobanks, with sample sizes ranging from 5023 to 1,320,016 individuals.

**Results:**

We found evidence of associations between genetically proxied SLC13A5 inhibition and higher creatinine-eGFR (*p* = 0.002), cystatin C-eGFR (*p* = 0.005), and lower BUN (*p* = 3 × 10^−4^). Statistical sensitivity analyses robust to the inclusion of pleiotropic variants suggested that these effects may be a consequence of higher plasma citrate levels. There was no strong evidence of associations of genetically proxied SLC13A5 inhibition with uACR or risk of CKD or microalbuminuria. Secondary analyses identified evidence of associations with higher plasma calcium levels (*p* = 6 × 10^−13^) and lower fasting glucose (*p* = 0.02). PheWAS did not identify any safety concerns.

**Conclusions:**

This Mendelian randomization analysis provides human-centric insight to guide clinical development of an SLC13A5 inhibitor. We identify plasma calcium and citrate as biologically plausible biomarkers of target engagement, and plasma citrate as a potential biomarker of mechanism of action. Our human genetic evidence corroborates evidence from various animal models to support effects of SLC13A5 inhibition on improving kidney function.

**Supplementary Information:**

The online version contains supplementary material available at 10.1186/s12916-023-03227-5.

## Background

The kidney is a highly metabolically active organ. The provision of citric acid cycle intermediates has ameliorated renal tubular damage and progression of chronic renal injury in animal models [1–3]. Solute carrier family 13 member 5 (SLC13A5), which is primarily expressed in hepatocytes of the liver, is a membrane-bound Na^+^-coupled co-transporter responsible for moving extracellular citrate into the cytosol [4, 5]. Systemic SLC13A5 inhibition could augment citrate flux to the kidney, thereby supporting renal energy metabolism. Moreover, given the role of citrate in regulating glucose metabolism, lipid metabolism, and inflammation, SLC13A5 inhibition could indirectly affect renal health [6–13]. However, no SLC13A5 inhibitor has yet entered clinical study, and so evidence of its efficacy for ameliorating progression of kidney disease in humans is limited. Additionally, SLC13A5 shows species-specific effects [11, 14–16].

Insights from human genetic data offer a powerful opportunity to prioritize and inform the design of clinical research. Given that learnings from such genetic analyses relate to the target organism, namely humans, they are well-placed to overcome some of the limitations of translating findings from animal models [17]. As genes code for proteins and proteins make up the majority of drug targets, it follows that naturally occurring variation in the genes coding for drug target proteins can be leveraged to inform on the effect of their pharmacological perturbation [18]. The random allocation of genetic variants at conception means that their association with clinical traits and phenotypes are less susceptible to the confounding factors and reverse causation bias that can hinder causal inference in traditional epidemiological study designs. The current availability of publicly accessible large-scale genetic association data also means that such a drug target Mendelian randomization paradigm is a relatively fast and cost-effective approach for inferring the potential clinical effects of perturbing drug targets [19].

Given the pre-clinical evidence supporting potential therapeutic applications of SLC13A5 inhibition [10, 12, 13, 20, 21], we sought to investigate its effects using drug target Mendelian randomization. By utilizing the known effect of SLC13A5 inhibition on increasing plasma citrate levels to identify genetic instruments [10, 22, 23], our primary objective was to investigate its effects on parameters of kidney function. In secondary analyses, we aimed to unravel potential mechanisms of action, as well as evidence of broader effects on glucose and lipid metabolism, and inflammation. Such insights would thus serve to inform and prioritize clinical development efforts for SLC13A5 inhibitors.

## Methods

### Study design overview

Genetic instruments for SLC13A5 inhibition were first identified as minimally correlated (*r*^2^ < 0.1) single-nucleotide polymorphisms (SNPs) within 200kB of the *SLC13A5* gene (chromosome 17, base position 6,588,032 to 6,616,886 on reference panel GRCh37/hg19 by Ensembl) that associate with plasma citrate levels at a genome-wide significance level (*p* < 5 × 10^−8^). The inhibition of SLC13A5 would decrease the transport of citrate from the extracellular to the intracellular compartment. Thus, variants in *SLC13A5* that are associated with increased levels of plasma citrate may represent greater inhibition of SLC13A5. A relatively relaxed pruning threshold was used to maximise statistical power, as the selected genetic variants are confined to only the *SLC13A5* gene region. As a sensitivity check, the primary analyses were repeated using a more stringent pruning threshold of *r*^2^ < 0.01. Instrument validity was tested by performing Mendelian randomization to explore associations with plasma calcium levels, whose levels are postulated to be increased with SLC13A5 inhibition due to reduced sequestration in the bone [24]. Such an association would also support plasma calcium as a biomarker of target engagement for SLC13A5 inhibition.

To explore potential mechanisms by which the variants selected as instruments for SLC13A5 inhibition may be exerting their effects, they were functionally annotated using the PhenoScanner resources (version 2) [25]. The same resource was used to explore potential pleiotropic associations of any of these variants, by testing for associations with any phenotypes at *p* < 5 × 10^−5^, which is the recommended significance threshold to correct for the 1490 tested traits.

Primary Mendelian randomization analyses were then performed to investigate the association of genetically proxied SLC13A5 inhibition with creatinine and cystatin C-based measures of estimated glomerular filtration rate (eGFR), blood urea nitrogen (BUN), urine albumin-creatinine-ratio (uACR), and risk of microalbuminuria and chronic kidney disease (CKD).

To investigate whether any effect of SLC13A5 inhibition on parameters of kidney function may be attributable to metabolic effects in the plasma that consequently affect kidney function, metabolome-wide Mendelian randomization of the plasma and urine was undertaken for SLC13A5 inhibition. Evidence of concordant effects of SLC13A5 inhibition on biomarkers measured in the plasma and urine would be consistent with metabolic effects resulting in compensatory mechanisms in the kidney, whereas evidence of contrasting effects in the plasma and urine would suggest a process specific to the kidney underlying the effects.

Secondary Mendelian randomization analyses were performed to explore the human genetic evidence for effects of SLC13A5 inhibition on biomarkers of glucose and lipid metabolism, and inflammation, namely plasma low-density lipoprotein cholesterol (LDLc), high-density lipoprotein cholesterol (HDLc), triglycerides, fasting glucose, interleukin 6 (IL6), and C-reactive protein (CRP). Finally, given that SLC13A5 is predominantly expressed in the liver and is involved in glucose and lipid metabolism [10, 12, 20, 26], we also investigated potential effects on liver fat.

In order to gain potential insight into mediating mechanisms and pathways implicated in the effects of SLC13A5 inhibition, we also performed a proteome-wide Mendelian randomization study. This could serve to identify protein mediators for the effect of SLC13A5, as well as potential novel biomarkers of target engagement. Finally, to investigate potential on-target adverse effects or novel indications, we performed a phenome-wide association study (PheWAS) [27].

To explore whether plasma citrate is a potential mediating mechanism for any associations identified, and thus could serve as a biomarker of mechanism of action, all analyses that considered genetically proxied SLC13A5 as the exposure were also repeated for genetically predicted plasma citrate levels, by selecting instruments from throughout the genome rather than confined to the *SLC13A5* gene region.

**Fig. 1:**
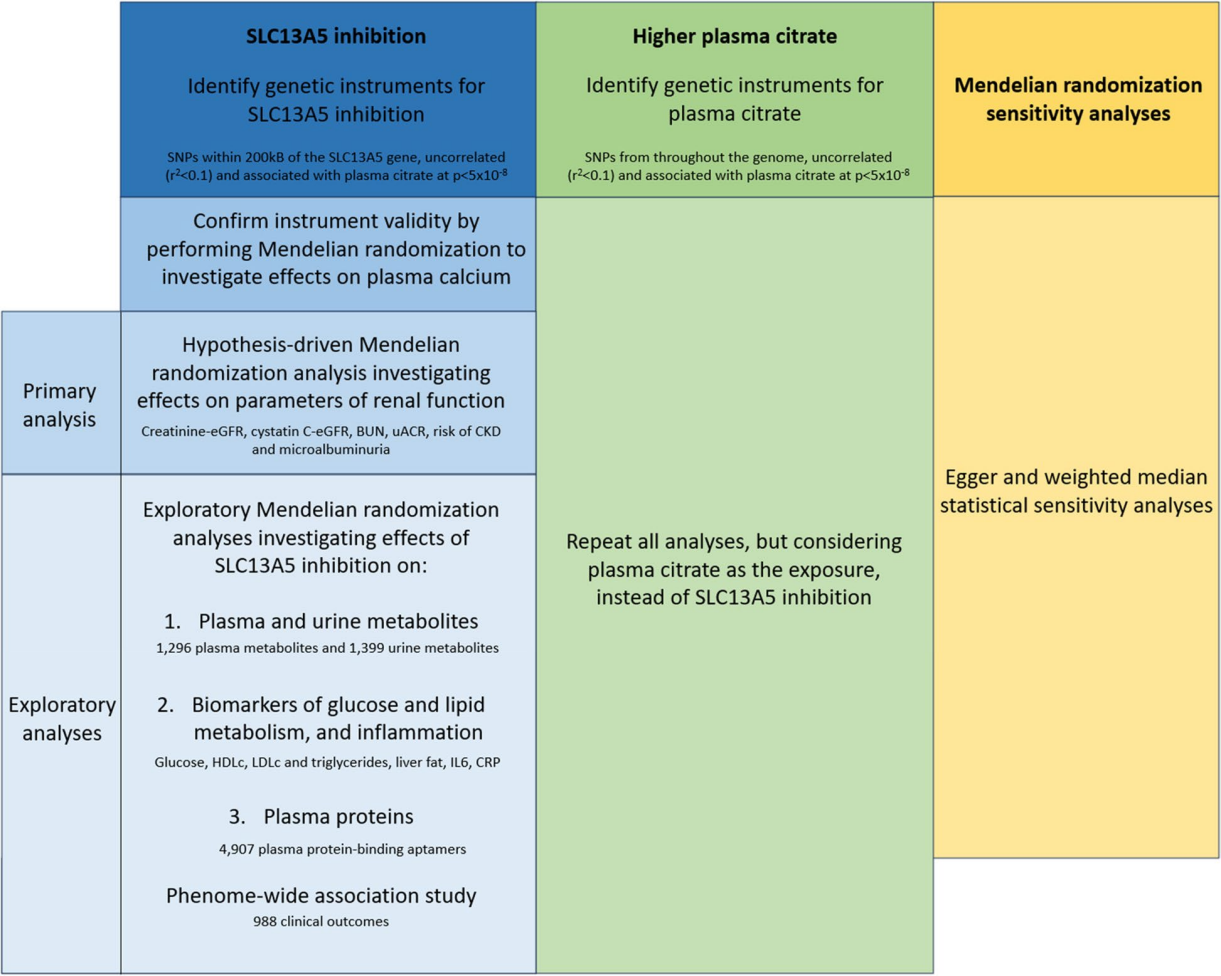
Overview of the study design. BUN: blood urea nitrogen, CRP:C-reactive protein, CKD: chronic kidney disease; eGFR: estimated glomerular filtration rate, HDLc: high-density lipoprotein cholesterol, IL6: interleukin 6; LDLc: low-density lipoprotein cholesterol, SNP: single-nucleotide polymorphism, uACR: urine albumin-creatinine ratio

A schematic figure depicting the overall study design is presented in Fig. 1.

### Statistical analysis

#### Instrument strength

Instrument strength was estimated by calculating the F-statistic for each SNP using the chi-squared approximation, which for each variant is the square of the SNP-exposure association estimate divided by the square of the SNP-exposure association estimate’s standard error [28].

#### Mendelian randomization

The genome-wide association study summary data sources used for the Mendelian randomization analyses are presented in Table 1 [29–39]. Participant consent and ethical approval for all data were obtained in the original studies.

**Table 1:** Genome-wide association study summary data used for the Mendelian randomization analyses

**Category**	**Trait**	**Unit**	**Population and measure**	**Sample size**	**Citation**
**Biomarker**	Plasma citrate	SD	European ancestry UK Biobank participants, using cross-sectional measurements	115,064	[29]
	Plasma calcium	SD	British ancestry UK Biobank participants, using cross-sectional measurements	361,194	[30]
**Renal**	Creatinine-eGFR	SD of log	Multi-ancestry GWAS meta-analysis, using cross-sectional measurements and formulae as described in the original study	1,201,929	[31]
	Cystatin C-eGFR	SD of log			
	Blood urea nitrogen	SD of log			
	Urine albumin-creatinine ratio	SD of log	Multi-ancestry GWAS meta-analysis, using spot measurements in a population including 51,541 individuals with diabetes	564,257	[32]
	Chronic kidney disease	Log OR	Multi-ancestry GWAS meta-analysis, with case definitions as those with cross-sectional eGFR measurements < 60 mL/min/1.73m^2^	625,219 (64,164 cases)	[33]
	Microalbuminuria	Log OR	Multi-ancestry GWAS meta-analysis, defined as urine albumin-creatinine ratio > 30 mg/g in spot measurements	348,954 (51,861 cases)	[32]
**Metabolome**	1296 plasma metabolites	SD	German Chronic Kidney Disease study, using cross-sectional measurements in individuals with eGFR 30-60 ml/min/1.73m^2^, or eGFR > 60 ml/min/1.73m^2^ with urine albumin-creatinine ratio > 300 mg/per g, or urinary protein/creatinine ratio > 500 mg/g	5023	[34]
	1399 urine metabolites	SD			
**Lipid, glucose and inflammation**	Plasma low-density lipoprotein cholesterol	SD	European ancestry GWAS meta-analysis, using cross-sectional measurements	1,320,016	[35]
	Plasma high-density lipoprotein cholesterol	SD			
	Plasma triglycerides	SD			
	Fasting plasma glucose	mmol/l	European ancestry GWAS meta-analysis, using cross-sectional measurements	200,622	[36]
	Imaging-derived liver fat (MRI-PDFF)	SD	UK Biobank participants, using cross-sectional measurements	36,703	[37]
	Plasma interleukin 6 binding aptamer level	SD	deCODE Icelandic study, using cross-sectional measurements	35,559	[38]
	Plasma C-reactive protein level	SD of log	European ancestry GWAS, using cross-sectional measurements	427,367	[39]
**Proteome**	4907 plasma protein-binding aptamers	SD	deCODE Icelandic study, using cross-sectional measurements	35,559	[38]

The full list of analysed metabolites and protein-binding aptamers are available in Additional file 2. Full study details and access to GWAS summary data are provided in the original publications. Exclusions were not made for individuals with polycystic kidney disease or renal stone disease. *BUN* blood urea nitrogen, *eGFR* estimated glomerular filtration rate, *GWAS* genome-wide association study, *MRI-PDFF* magnetic resonance imaging-derived proton density fat fraction, *OR* odds ratio, *SD* standard deviation, *uACR* urine albumin-creatinine ratio

The main Mendelian randomization analyses were performed using the random-effects inverse-variance weighted (IVW) method [40]. This meta-analyses the Wald ratio estimates for each instrument SNP using a random-effects inverse-variance model. After harmonising SNPs by their effect alleles, the Wald ratio estimate is calculated by dividing the SNP-exposure association estimate by the SNP-outcome association estimate. The standard error of the Wald ratio estimate was calculated using the propagation of error method.

Two Mendelian randomization statistical sensitivity analyses were employed, Egger and weighted median, which make distinct assumptions regarding the inclusion of pleiotropic variants. The Egger method regresses the SNP-outcome association estimates on the SNP-exposure association estimates [41]. The slope of the regression provides a Mendelian randomization estimate that is corrected for pleiotropic associations of the genetic variants and the intercept serves as a test for the presence of such pleiotropic associations, provided that the magnitudes of these pleiotropic associations are not correlated to instrument strength. The weighted median method orders the Wald ratio estimates for all instrument SNPs weighted by their precision, and selects the median value, with 95% confidence intervals (95% CIs) calculated by bootstrapping [42]. It provides an accurate Mendelian randomization estimate when more than half the information for the analysis comes from valid instruments. Heterogeneity in MR estimates derived from individual SNPs can also signify the presence of potential bias from pleiotropy and was assessed using Cochran’s Q test [43]. All Mendelian randomization analyses were performed using the “MendelianRandomization” package for R statistical software [44].

#### Colocalisation analysis

For any primary outcomes where there was Mendelian randomization evidence of an effect of SLC13A5 inhibition, we performed colocalisation analysis using a 200-kB window around the SLC13A5 gene, as detailed above [45]. This analysis aims to test whether there is a shared causal variant underlying any observed Mendelian randomization association, which would support a causal effect of SLC13A5 inhibition on the outcome [46].

#### Phenome-wide association study

PheWAS was performed in the UK Biobank, a prospective cohort study initiated in 2006 that recruited more than 500,000 participants aged between 40 and 69 years [47]. Participants contributed phenotypic and genetic data, as well as biological samples, as previously reported [48]. UK Biobank has approval from the North West Multi-centre Research Ethics Committee and all participants provided appropriate consent.

PheWAS statistical analysis was performed by first constructing a weighted genetic risk score for the exposure using the same instrument SNPs as employed in the Mendelian randomization analysis. For each individual included in the analysis, the number of plasma citrate-increasing alleles for each instrument SNP was multiplied by the corresponding genetic association estimate with citrate for that SNP, before combining by addition. International Classification of Diseases versions 9 and 10 were used to ascertain cases in the UK Biobank Hospital Episode Statistics data, with diagnoses linked to the phenotype code (phecode) grouping system to facilitate classification of clinically relevant traits [49]. Logistic regression was performed for each phecode against the standardized genetic risk score for the exposure, adjusting for age, sex, and the first 10 genetic principal components of genetic ancestry. Only phecodes with 200 or more cases were included in the analysis, to avoid inclusion of outcomes with low statistical power. PheWAS estimates are reported per 1-SD higher standardized exposure genetic risk score. PheWAS was conducted using the “PheWAS” package of R statistical software [50].

#### Statistical significance ascertainment

To account for testing of multiple outcomes in the primary Mendelian randomization analysis of renal outcomes, the Benjamini–Hochberg false discovery rate (FDR) 5% threshold was used. No statistical significance threshold was used for the other analyses, which were exploratory in nature. All presented *p* values are uncorrected for multiple testing, unless otherwise stated.

#### Comparison of SLC13A5 inhibition with higher plasma citrate levels by any mechanism

The Mendelian randomization and PheWAS analyses were also performed considering the exposure of plasma citrate levels, rather than SLC13A5 inhibition. Selection of genetic variants to serve as instruments and build the weighted genetic risk score for plasma citrate was undertaken using the same approach as for SLC13A5 inhibition, except that selection of variants was not confined to the *SLC13A5* gene region.

#### Availability of code and data

R statistical software was used for all analyses. The statistical code used in this work is available upon reasonable request to the corresponding author. All genome-wide association study summary data are publicly available from the sources cited in Table 1. UK Biobank individual participant data are available upon appropriate application to the UK Biobank study. This work was reported using the “Strengthening the Reporting of Observational Studies in Epidemiology using Mendelian Randomization” (STROBE-MR) checklist (Additional file 1) [51].

## Results

A total of 13 SNPs were identified as instruments for SLC13A5 inhibition (Additional file 2: Table S1), all of which had F-statistics > 10, consistent with low risk of weak instrument bias that might affect the conclusions of Mendelian randomization analyses. Functional annotations of the SNPs obtained using PhenoScanner version 2 are provided in Additional file 2: Table S2. There were only potential pleiotropic associations of one variant (rs75448233) with smoking traits; however, these associations did not reach genome-wide significance (Additional file 2: Table S3).

The main IVW Mendelian randomization identified positive associations of genetically proxied SLC13A5 inhibition with plasma calcium levels. Every 1-SD higher genetically proxied plasma citrate through SLC13A5 inhibition was associated with a 0.132 SD units higher plasma calcium level (95% CI 0.107 to 0.157, *p* = 6 × 10^−13^).

**Fig. 2:**
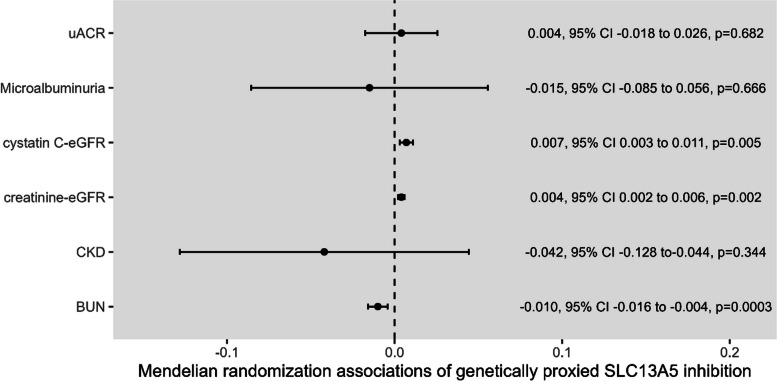
Mendelian randomization estimates for the association of genetically proxied SLC13A5 inhibition with kidney traits. Mendelian randomization estimates are scaled per 1-standard deviation (SD) increase in plasma citrate through genetically proxied SLC13A5 inhibition. The units for the BUN, creatinine-eGFR, cystatin C-eGFR and uACR are SD change in log transformed levels. The units for CKD and microalbuminuria are log odds ratio. Raw *p* values are presented. BUN: blood urea nitrogen, chronic kidney disease; eGFR: estimated glomerular filtration rate, urine albumin-creatinine ratio

Figure 2 depicts the associations of genetically proxied SLC13A5 inhibition with kidney function parameters from the main IVW Mendelian randomization analysis. There were statistically significant associations of genetically proxied SLC13A5 inhibition with higher creatinine and cystatin C-based measures of eGFR (FDR adjusted *p* values = 0.006 and 0.010 respectively), and lower BUN (FDR adjusted *p* value = 0.002). There were no strong associations of genetically proxied SLC13A5 inhibition with uACR (FDR adjusted *p* value = 0.682), or risk of CKD (FDR adjusted *p* value = 0.516) or microalbuminuria (FDR adjusted *p* value = 0.682). Additional file 3: Fig. S1-S6 show scatter plots of the SNP-exposure and SNP-outcome associations. Similar Mendelian randomization point estimates were obtained when using a pruning threshold of r^2^ < 0.01 (Additional file 2: Table S4).

Results of the colocalisation analysis for primary outcomes showing significant Mendelian randomization associations are presented in Additional file 2: Table S5. For all these analyses, there was only evidence of a causal variant in the locus affecting plasma citrate levels, suggesting that there was inadequate statistical power to test whether the Mendelian randomization associations were attributable to shared or distinct causal variants [46].

The results of the metabolome-wide Mendelian randomization analysis investigating the effects of SLC13A5 inhibition on plasma and urine metabolites are presented in Additional file 2: Table S6 and S7. As expected, there was genetic evidence supporting associations of SLC13A5 inhibition with higher plasma citrate. Additionally, there was evidence supporting associations of SLC13A5 inhibition with higher plasma aconitate and plasma N-acetylspermidine.

**Fig. 3:**
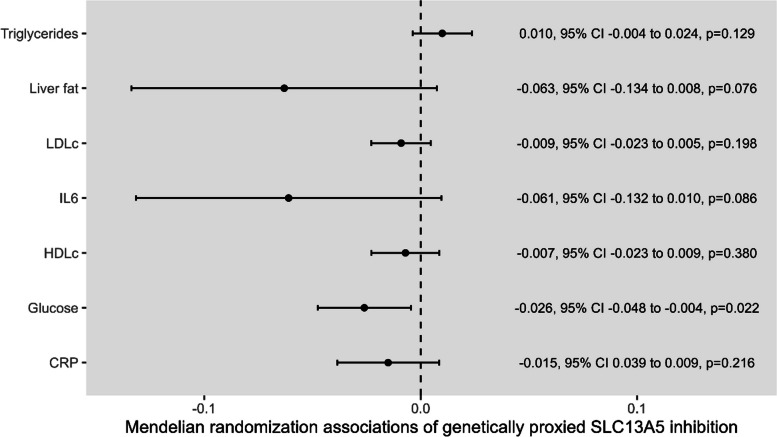
Mendelian randomization estimates for the association of genetically proxied SLC13A5 inhibition with biomarkers of glucose and lipid metabolism, and inflammation. Mendelian randomization estimates are scaled per 1-standard deviation (SD) increase in plasma citrate through genetically proxied SLC13A5 inhibition. The units for fasting glucose is mmol/l, the units for C-reactive protein (CRP) is standard deviation (SD) change of log transformed levels, and the remaining exposures are measured in SD units. HDLc: high-density lipoprotein cholesterol, IL6: interleukin 6, LDLc: low-density lipoprotein cholesterol

The main IVW Mendelian randomization analysis results measuring the association of genetically proxied SLC13A5 inhibition with parameters of glucose and lipid metabolism, and inflammation are presented in Fig. 3. In this hypothesis-generating analysis, there were no significant associations after adjusting for multiple testing, but some evidence to support possible associations of SLC13A5 inhibition with lower fasting glucose levels (− 0.026 mmol/l per 1-SD higher plasma citrate through SCL13A5 inhibition, 95% CI − 0.047 to 0.004, raw *p* = 0.022) [10, 21]. Neither proteome-wide Mendelian randomization (Additional file 2: Table S8) nor PheWAS (Additional file 2: Table S9) identified any significant associations for genetically proxied SLC13A5 inhibition after correcting for multiple testing.

All analyses were also performed considering higher plasma citrate through any mechanism as the exposure, instead of genetically proxied SLC13A5 inhibition (i.e., SNPs associated with higher plasma citrate selected as in the analysis for SLC13A5, but not restricted to 200kB within the *SLC13A5* gene). The SNPs used as instruments for plasma citrate are presented in Additional file 2: Table S10, and the analysis results are presented in Additional file 2: Tables S11-S14. Although the main IVW analysis did not identify evidence supporting associations of genetically predicted plasma citrate levels with eGFR or BUN, the Egger and weighted median sensitivity analyses that are more robust to the inclusion of pleiotropic SNPs were more supportive of such an association (Additional file 2: Table S15). This suggests that the genetic variants that affect plasma citrate are heterogeneous and pleiotropic in the mechanisms by which they achieve effects on eGFR and BUN. Consistent with this, there was strong evidence of heterogeneity in all the IVW MR analyses undertaken with plasma citrate as the exposure (Additional file 2: Table S15). This implies that increasing plasma citrate through SLC13A5 inhibition would potentially improve eGFR and BUN, but that increasing plasma citrate by other mechanism not necessarily achieve this same result.

## Discussion

Using large-scale genetic association data, we identified a genetic instrument for SLC13A5 inhibition that we leveraged in the Mendelian randomization paradigm to provide clinically translatable insights. Our main findings generated human genetic evidence that supports higher plasma citrate and calcium as biologically plausible biomarkers of target engagement, higher plasma citrate as a potential mechanism of action biomarker, and favourable effects on parameters of kidney function, namely creatinine and cystatin C-based measures of eGFR, and BUN. Of note, we did not identify statistically significant evidence of effects of SLC13A5 inhibition on uACR nor risk of microalbuminuria. This discrepancy may be explained by these biomarkers measuring different domains of kidney function [52]. Of relevance, calcium citrate treatment in an acute kidney damage animal model resulted in significantly reduced levels of proteinuria [1]. The primary Mendelian randomization analysis also did not identify evidence of SLC13A5 inhibition having effects on CKD risk, which may in part be explained by the pathophysiological heterogeneity underlying CKD [53], and SLC13A5 inhibition potentially only being relevant to some of these mechanisms.

Secondary Mendelian randomization analyses supported potential effects of SLC13A5 inhibition on reducing fasting glucose. Given that SLC13A5 is predominantly expressed in the liver [26], these findings are consistent with direct hepatic effects. The lack of strong associations of genetically proxied SLC13A5 inhibition with clinical outcomes in PheWAS is reassuring regarding safety profile, although the limited statistical power also means that findings may warrant further investigation. This suggests smaller reductions in SLC13A5 activity are unlikely to recapitulate the autosomal-recessive epileptic encephalopathy phenotype observed with rare mutations affecting critical regions of the *SLC13A5* gene [54].

SLC13A5 is a membrane-bound citrate transporter, and its inhibition results in reduced entry of plasma citrate into the cytosol [4, 5]. This would be expected to have implications for cellular metabolism in cells expressing SLC13A5, with the higher circulating plasma citrate levels also potentially impacting metabolism systemically. Consistent with the results of our main genetic analyses, previous work has identified protective effects of citrate in various animal models of kidney disease [1, 6, 55, 56]. Although the main IVW Mendelian randomization analysis investigating effects of plasma citrate levels did not identify evidence supporting effects on parameters of kidney function, there was strong evidence of heterogeneity in estimates generated by individual SNPs, consistent with the presence of pleiotropic effects that could bias the main IVW analyses [43]. Furthermore, the Egger and weighted median Mendelian randomization statistical sensitivity analyses, which can be more robust to the inclusion of pleiotropic variants, produced estimates more suggestive of favourable effects of higher plasma citrate on measures of kidney function. This observation may suggest that plasma citrate is a heterogeneous trait that is affected through several distinct pathways, and that at least some of these may favourably impact kidney function. This would be in-keeping with the favourable effects of SLC13A5 on kidney function occurring through its effects on plasma citrate. It has been reported that low citrate levels in urine are associated with various forms of kidney disease, and low urinary citrate is a biomarker for kidney disease progression [55, 57–62]. Furthermore, pre-clinical studies with SLC13A5 knockout mice have supported effects on sympathoadrenal mechanisms [63], which may also contribute to nephroprotective effects of genetically proxied SLC13A5 inhibition.

There are several hypotheses for how citrate may exert its beneficial effects on kidney function. Generally, elevated plasma citrate levels are proposed to lead to increased excretion of citrate through the urine. Higher urinary citrate may in turn provide an additional energy supply for kidney cells [1–3], potentially enhancing their resilience and function. Furthermore, citrate may contribute to supporting the biosynthesis of essential molecules such as glucose, lipids, and amino acids. Additionally, it is known, due to its alkalinizing function, citrate contributes to improved acid–base balance within the kidneys, promoting a favourable environment for cellular processes. Finally, urinary citrate’s solubilizing properties might offer protection against vascular calcification and the formation of kidney stones, thus safeguarding kidney health.

This work has several strengths. By leveraging large-scale genetic association data within the Mendelian randomization paradigm, we were able to rapidly perform several hypothesis-driven and hypothesis-free genetic analyses relevant to humans, and consequently generate insight related to drug development. Our findings represent numerous potential learnings, including highlighting higher plasma citrate and calcium as biomarkers of target engagement, higher plasma citrate as a biomarker of mechanism of action, efficacy for improving kidney function, general safety profile, and potential effects on reducing fasting glucose. Further, the random allocation of genetic variants at conception means that these findings are less vulnerable to the confounding and reverse causation bias that can hinder causal inference in traditional epidemiological study designs [64].

This work should also be interpreted in the context of its limitations. The Mendelian randomization paradigm considers small lifelong effects of germline genetic variation. This is not the same as a discrete clinical intervention in later life, which may be of shorter duration but larger magnitude. Thus, it is possible that some of the findings of these genetic analyses may not predict what is observed with inhibition of SLC13A5 in clinical practice, particularly in quantitative terms. Further, some of our current analyses may also be limited by phenotypic definition and statistical power. For example, we note that kidney traits were not prioritised in the results of PheWAS analysis. In addition, the colocalization analyses did not support a single causal variant underlying the observed Mendelian randomization associations, and this may be attributable to either low statistical power, or confounding due to variant in linkage disequilibrium. In the Mendelian randomization analyses, we used a combination of European ancestry and multi-ancestry genetic association data, creating the potential for bias related to population substructure. Similarly, there may be interactions of the genetic variants employed as instruments with dietary intake of citrate, which may vary across population groups, potentially also introducing bias. While challenges in estimating the variance of drug target perturbation predicted by genetic variants means that it is not possible to perform conventional power calculations for drug target Mendelian randomization [65], some indication of the relative statistical power available for each analysis is apparent from the size of the 95% CIs. From this, it is clear that the analyses considering CKD had much less power than those considering measures of eGFR or BUN, for example. Of relevance, numerous preclinical mechanistic studies have demonstrated a role of SLC13A5 in favourably affecting hepatic lipid and glucose metabolism [10–13, 20, 21, 66, 67], and the limitations of our current analyses may explain the discrepancy in findings. The plasma and urine metabolome-wide Mendelian randomization analyses were undertaken using outcome data obtained from a CKD population and may thus be vulnerable to collider bias [68]. This is because stratifying on a collider can introduce associations with confounding factors. Our Mendelian randomization evidence supports effects of SLC13A5 inhibition on parameters of kidney function, in-keeping with CKD therefore representing a potential collider. While there are strategies available to help explore potential bias from this [69], they either require individual participant data, which were not available for the current Mendelian randomization analyses, or have other prohibitive limitations [70]. Finally, the genetic analyses undertaken in this work provide insight into the on-target effects of perturbing SLC13A5 [19], but cannot directly inform on the effects of any pharmacological agents used to inhibit SLC13A5, including their pharmacokinetic and pharmacodynamic properties, or off-target effects.

## Conclusions

In summary, this Mendelian randomization analysis provides novel human-centric insight to guide the clinical development of an SLC13A5 inhibitor. We identify biologically plausible biomarkers of target engagement and mode of action, as well as genetic evidence supporting potential effects in improving kidney function, with higher plasma citrate levels a possible underlying mechanism. The null PheWAS findings are reassuring in that they do not identify any safety signals. Further study in the form of early-stage clinical trials may now be warranted to help translate these findings towards improving patient care.

### Supplementary Information

**Additional file 1.** Strengthening the Reporting of Observational Studies in Epidemiology using Mendelian Randomization checklist.

**Additional file 2: Table S1.** Single-nucleotide polymorphisms used as instruments for SLC13A5 inhibition. Genetic associations with plasma citrate are presented for variants within 200kB of the *SLC13A5* gene. SNP: single-nucleotide polymorphism. **Table S2.** Annotation of the functional consequences of the single-nucleotide polymorphisms used to instruments SLC13A5 inhibition using the PhenoScanner version 2 database. **Table S3.** Associations of the variants employed as instruments for SLC13A5 inhibition in the PhenoScanner version 2 database below the recommended threshold of*P*<5x10-5, which accounts for multiple testing of the 1490 included genome-wide association studies. **Table S4.** Inverse-variance weighted Mendelian randomization sensitivity analyses using a pruning correlation threshold of r2<0.01 for variants employed as instruments. The exposure is genetically proxied SLC13A5 inhibition, as in the main analysis. This sensitivity analysis is performed for the primary outcomes, related to kidney traits. **Table S5.** Results of the genetic colocalisation analysis. H0 signifies the probability that there is no genetic association in the SLC13A5 gene region with either plasma citrate or the considered outcome. H1 signifies the probability that there is only an association with plasma citrate. H2 signifies the probability that there is only an association with the outcome. H3 signifies the probability that there is an association with plasma citrate and the outcome that is attributable to distinct causal variants. H4 signifies the probability that there is an association with plasma citrate and the outcome that is attributable to a shared causal variant. **Table S6.** Metabolome-wide Mendelian randomization analysis investigating the associations of genetically proxied SLC13A5 inhibition with plasma metabolites. CI: confidence interval. **Table S7.** Metabolome-wide Mendelian randomization analysis investigating the associations of genetically proxied SLC13A5 inhibition with urine metabolites. CI: confidence interval. **Table S8.** Proteome-wide Mendelian randomization analysis investigating the associations of genetically proxied SLC13A5 inhibition with plasma protein-binding aptamers. **Table S9.** Phenome-wide association study investigating the associations of a standardised genetic risk score for SLC13A5 inhibition with clinical outcomes across the phenome. **Table S10.** Single-nucleotide polymorphisms used as instruments for plasma citrate levels, selected from throughout the genome. SNP: single-nucleotide polymorphism. **Table S11.** Metabolome-wide Mendelian randomization analysis investigating the associations of genetically predicted plasma citrate levels with plasma metabolites. CI: confidence interval. **Table S12.** Metabolome-wide Mendelian randomization analysis investigating the associations of genetically predicted plasma citrate levels with urine metabolites. CI: confidence interval. **Table S13.** Proteome-wide Mendelian randomization analysis investigating the associations of genetically predicted plasma citrate levels with plasma protein-binding aptamers. **Table S14.** Phenome-wide association study investigating the associations of a standardised genetic risk score for plasma citrate with clinical outcomes across the phenome. **Table S15.** Mendelian randomization statistical sensitivity analyses for biomarkers of renal function, glucose and lipid metabolism, and inflammation.

**Additional file 3: Figure S1.** A scatter plot of genetic association estimates for the SLC13A5 inhibition instrument variants with plasma citrate (x-axis) and blood urea nitrogen (BUN, y-axis). **Figure S2.** A scatter plot of genetic association estimates for the SLC13A5 inhibition instrument variants with plasma citrate (x-axis) and chronic kidney disease (CKD) risk (y-axis). **Figure S3.** A scatter plot of genetic association estimates for the SLC13A5 inhibition instrument variants with plasma citrate (x-axis) and creatine-based estimated glomerular filtrate rate (eGFR, y-axis). **Figure S4.** A scatter plot of genetic association estimates for the SLC13A5 inhibition instrument variants with plasma citrate (x-axis) and cystatin C-based estimated glomerular filtrate rate (eGFR, y-axis). **Figure S5.** A scatter plot of genetic association estimates for the SLC13A5 inhibition instrument variants with plasma citrate (x-axis) and microalbuminuria risk (y-axis). **Figure S6.** A scatter plot of genetic association estimates for the SLC13A5 inhibition instrument variants with plasma citrate (x-axis) and urine albumin-creatinine ratio (UACR, y-axis).

## Data Availability

R statistical software was used for all analyses. The statistical code used in this work is available upon reasonable request to the corresponding author. All genome-wide association study summary data are publicly available from the sources cited in Table 1. UK Biobank individual participant data are available upon appropriate application to the UK Biobank study.

## Abbreviations

BUN: Blood urea nitrogen
CI: Confidence interval
CKD: Chronic kidney disease
CRP: C-reactive protein
eGFR: Estimated glomerular filtration rate
FDR: False discovery rate
GWAS: Genome-wide association study
HDLc: High-density lipoprotein cholesterol
IL6: Interleukin-6
IVW: Inverse-variance weighted
LDLc: Low-density lipoprotein cholesterol
OR: Odds ratio
PheWAS: Phenome-wide association study
SD: Standard deviation
SNP: Single-nucleotide polymorphism
uACR: Urine albumin-creatinine ratio
