# Supplementary material for: The citrate transporter SLC13A5 as a therapeutic target for kidney disease: evidence from Mendelian randomization to inform drug development
Source: BMC Med. 2023 Dec 18;21:504. doi: 10.1186/s12916-023-03227-5 (PMC10729503; doi:10.1186/s12916-023-03227-5)
Supplement: Supplementary file 3 — Additional file 3: Figure S1. A scatter plot of genetic association estimates for the SLC13A5 inhibition instrument variants with plasma citrate (x-axis) and blood urea nitrogen (BUN, y-axis). Figure S2. A scatter plot of genetic association estimates for the SLC13A5 inhibition instrument variants with plasma citrate (x-axis) and chronic kidney disease (CKD) risk (y-axis). Figure S3. A scatter plot of genetic association estimates for the SLC13A5 inhibition instrument variants with plasma citrate (x-axis) and creatine-based estimated glomerular filtrate rate (eGFR, y-axis). Figure S4. A scatter plot of genetic association estimates for the SLC13A5 inhibition instrument variants with plasma citrate (x-axis) and cystatin C-based estimated glomerular filtrate rate (eGFR, y-axis). Figure S5. A scatter plot of genetic association estimates for the SLC13A5 inhibition instrument variants with plasma citrate (x-axis) and microalbuminuria risk (y-axis). Figure S6. A scatter plot of genetic association estimates for the SLC13A5 inhibition instrument variants with plasma citrate (x-axis) and urine albumin-creatinine ratio (UACR, y-axis). [file 12916_2023_3227_MOESM3_ESM.docx]

**Additional file 3**

**The citrate transporter SLC13A5 as a therapeutic target for kidney disease: evidence from Mendelian randomization to inform drug development**

**Dipender Gill, Loukas Zagkos, Rubinder Gill, Thomas Benzing, Jens Jordan, Andreas L. Birkenfeld, Stephen Burgess, Grit Zahn**


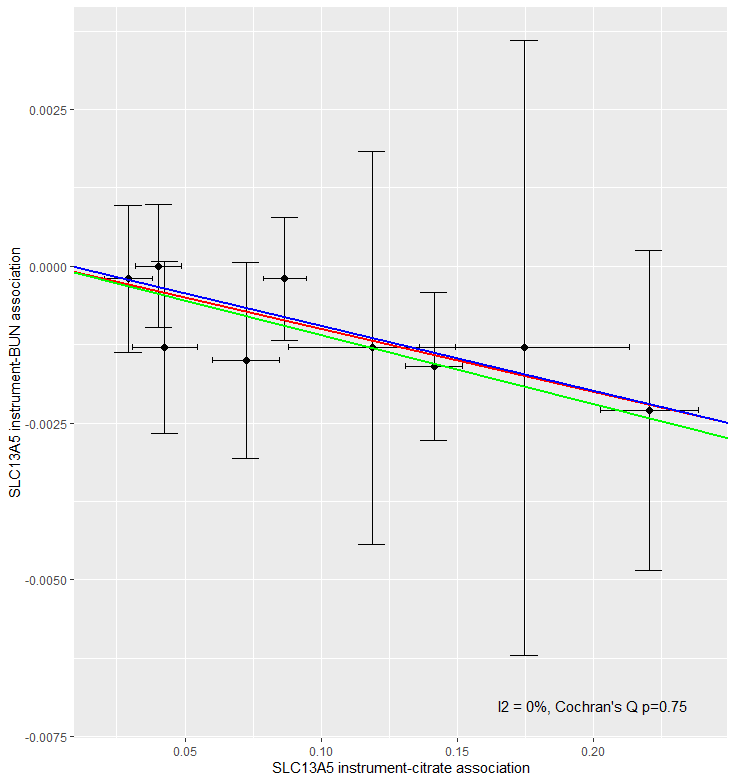


Figure S1. A scatter plot of genetic association estimates for the SLC13A5 inhibition instrument variants with plasma citrate (x-axis) and blood urea nitrogen (BUN, y-axis). The red line depicts the inverse-variance weighted Mendelian randomization analysis (estimate -0.010, standard error 0.003), the blue line depicts the Egger analysis (estimate -0.010, standard error 0.005, intercept estimate 8x10^-5^, intercept p=0.857), and the green line depicts the weighted median analysis (estimate -0.011, standard error 0.003).


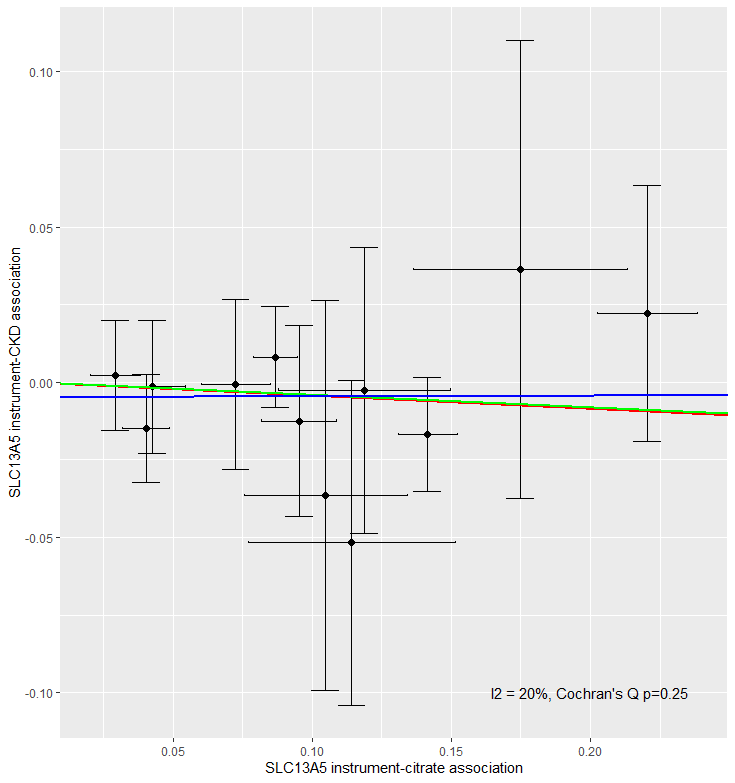


Figure S2. A scatter plot of genetic association estimates for the SLC13A5 inhibition instrument variants with plasma citrate (x-axis) and chronic kidney disease (CKD) risk (y-axis). The red line depicts the inverse-variance weighted Mendelian randomization analysis (estimate -0.042, standard error 0.044), the blue line depicts the Egger analysis (estimate 0.002, standard error 0.090, intercept estimate -0.005, intercept p=0.569), and the green line depicts the weighted median analysis (estimate -0.040, standard error 0.057).


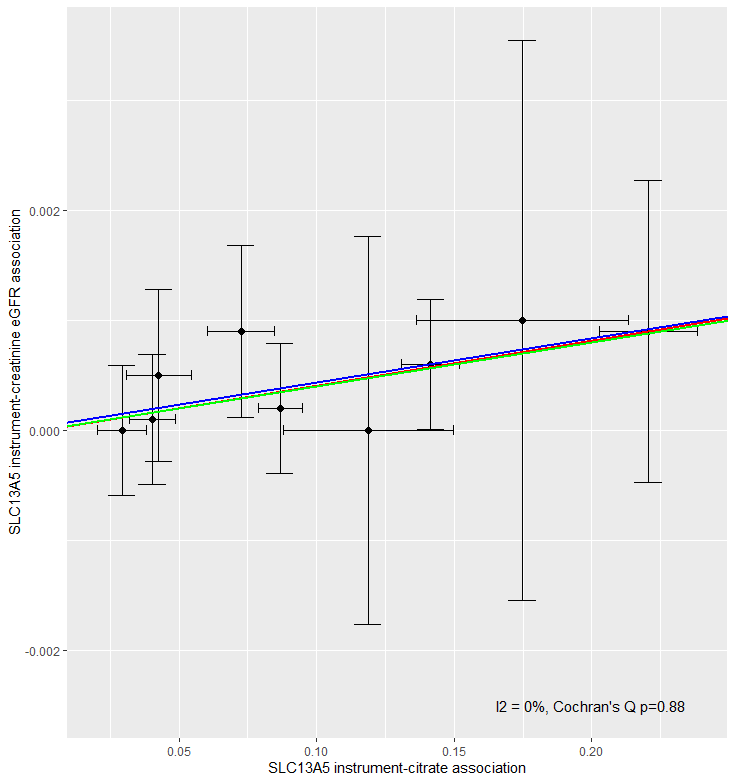


Figure S3. A scatter plot of genetic association estimates for the SLC13A5 inhibition instrument variants with plasma citrate (x-axis) and creatine-based estimated glomerular filtrate rate (eGFR, y-axis). The red line depicts the inverse-variance weighted Mendelian randomization analysis (estimate 0.004, standard error 0.001), the blue line depicts the Egger analysis (estimate 0.004, standard error 0.003, intercept estimate 3x10^-5^, intercept p=0.884), and the green line depicts the weighted median analysis (estimate 0.004, standard error 0.002).


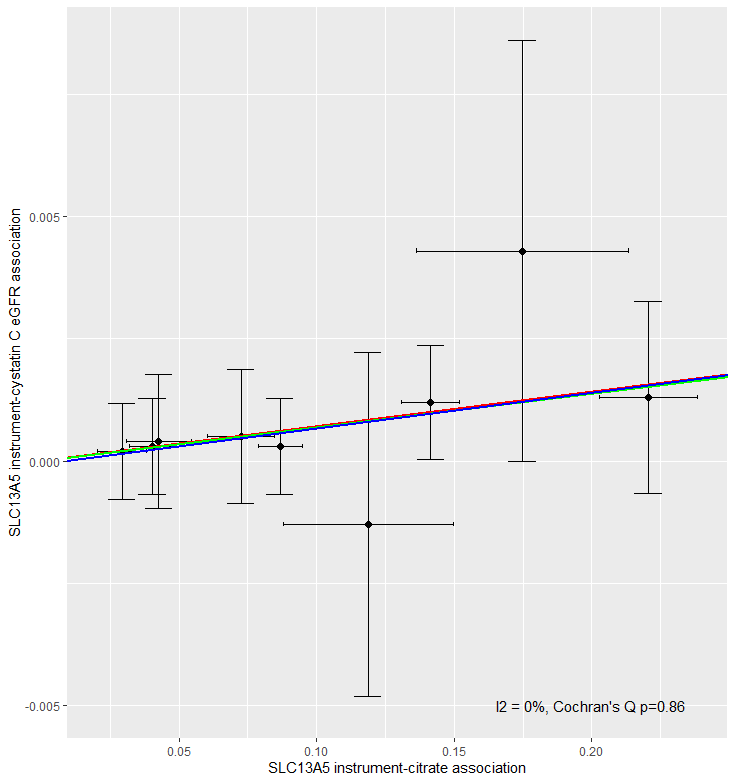


Figure S4. A scatter plot of genetic association estimates for the SLC13A5 inhibition instrument variants with plasma citrate (x-axis) and cystatin C-based estimated glomerular filtrate rate (eGFR, y-axis). The red line depicts the inverse-variance weighted Mendelian randomization analysis (estimate 0.007, standard error 0.002), the blue line depicts the Egger analysis (estimate 0.007, standard error 0.004, intercept estimate -6x10^-5^, intercept p=0.889), and the green line depicts the weighted median analysis (estimate 0.007, standard error 0.003).


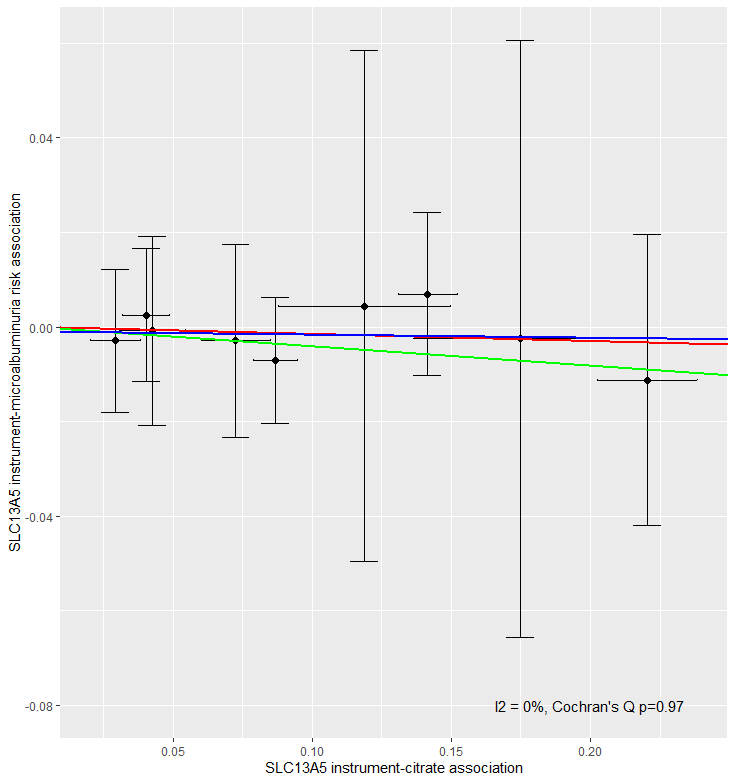


Figure S5. A scatter plot of genetic association estimates for the SLC13A5 inhibition instrument variants with plasma citrate (x-axis) and microalbuminuria risk (y-axis). The red line depicts the inverse-variance weighted Mendelian randomization analysis (estimate -0.015, standard error 0.036), the blue line depicts the Egger analysis (estimate -0.001, standard error 0.006, intercept estimate -0.001, intercept p=0.871), and the green line depicts the weighted median analysis (estimate -0.041, standard error 0.044).


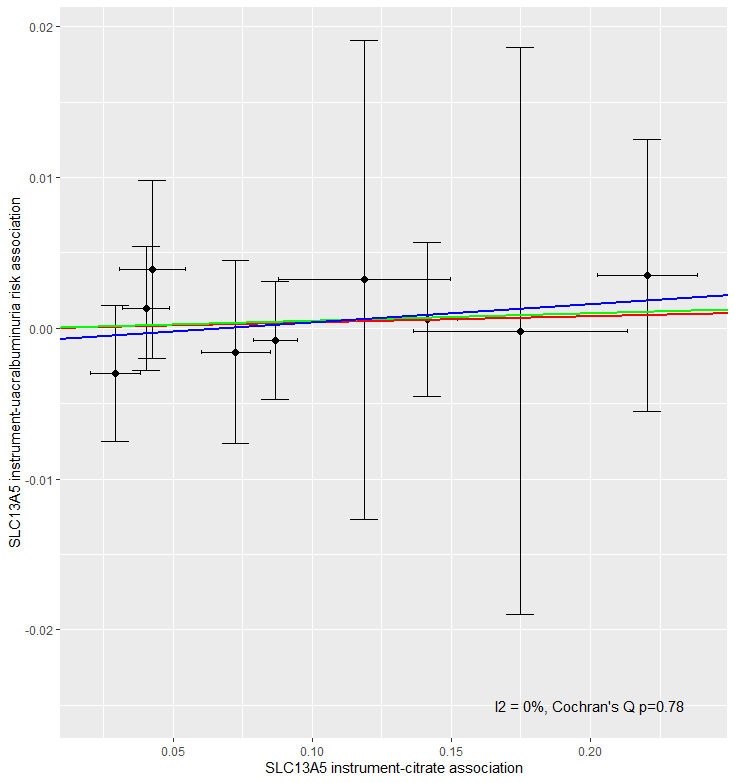


Figure S6. A scatter plot of genetic association estimates for the SLC13A5 inhibition instrument variants with plasma citrate (x-axis) and urine albumin-creatinine ratio (UACR, y-axis). The red line depicts the inverse-variance weighted Mendelian randomization analysis (estimate 0.004, standard error 0.011), the blue line depicts the Egger analysis (estimate 0.012, standard error 0.02, intercept estimate -0.001, intercept p=0.638), and the green line depicts the weighted median analysis (estimate 0.005, standard error 0.013).
